# Therapeutic Management of Chronic Lymphocytic Leukemia Presenting with Recurrent Massive Ascites

**DOI:** 10.3390/curroncol29100534

**Published:** 2022-09-22

**Authors:** Ugochi Ebinama, Nathaniel R. Wilson, Anindita Ghosh, Binsah S. George

**Affiliations:** 1 Department of Internal Medicine, McGovern Medical School, The University of Texas Health Sciences Center at Houston, Houston, TX 77030, USA; 2 Department of Pathology and Laboratory Medicine, McGovern Medical School, The University of Texas Health Sciences Center at Houston, Houston, TX 77030, USA; 3 Division of Hematology/Oncology, McGovern Medical School, The University of Texas Health Sciences Center at Houston, Houston, TX 77030, USA

**Keywords:** chronic lymphocytic leukemia, ascitic fluid with CLL, isolated ascitic fluid CLL, peritoneal fluid CLL

## Abstract

Chronic lymphocytic leukemia (CLL) is a lymphoproliferative malignancy that is categorized by the production and accumulation of CD5+ monoclonal B cell lymphocytes, commonly in the spleen, bone marrow, and peripheral blood; these are morphologically mature lymphocytes with abnormal immune function. Ascites, although common in solid organ malignancies such as ovarian, breast, and gastrointestinal, is a rare clinical manifestation in hematological malignancies. The case presented herein describes an elderly male patient with CLL who presented with transudative ascites 7 years after the completion of chemotherapy. Microscopic analysis and flow cytometry of the patient’s ascitic fluid were consistent with CLL, and he was treated with six cycles of obinutuzumab immunotherapy with the addition of acalabrutinib, resulting in near resolution of malignant ascites. A few cases have reported CLL manifesting as transudative or exudative ascites in elderly patients. A few previous cases have reported the development of ascites between 12 and 21 months after the initial treatment of CLL with chemotherapy. A unique feature of our patient is the presentation with malignant ascites nearly 7 years after the initial CLL treatment with chemotherapy. The intent of this case report is to bring awareness of ascites as a possible initial presenting symptom of CLL in patients with isolated abdominal distention with or without common clinical features of leukemia (i.e., splenomegaly, lymphadenopathy, and B-symptoms) and the therapeutic management thereafter. Malignant ascites may be associated with relapse or the transformation of leukemia; thus, prompt diagnosis and treatment should not be delayed.

## 1. Introduction

The diagnosis of CLL involves the presence of lymphocytosis (>5000 K) in addition to unique clonal immune phenotypes on peripheral blood flow cytometry. The pathogenesis of CLL involves the continuous activation of CD5+ B cells by genetic mutations, cytogenetic dysfunction, and environmental factors. An important step in the pathogenesis of CLL is the expression of B cell antigen receptor (BCR), which leads to continuous antigen-independent cell-autonomous cell signaling and thus to the production of malignant B lymphocytes (MBLs). The neoplastic B lymphocytes evade apoptosis and, with time, proliferate in the lymph nodes, eventually accumulating in peripheral blood. These MBLs may infiltrate the spleen and bone marrow, leading to splenomegaly and hypercellular bone marrow [1].

The primary sites of involvement for CLL include peripheral blood, bone marrow, spleen, and lymph nodes [2]. The pathologic leukemic phase is characterized by the presence of dysfunctional B cell lymphocytes in the peripheral blood smear. The most common clonal immunophenotypic expression seen in flow cytometry for CLL includes CD5, CD19, and CD23, in addition to the expression of one type of immunoglobulin light chain [3]. High-risk cytogenetic markers using fluorescent in situ hybridization (FISH) on peripheral blood include del17p, del11q, and unmutated immunoglobulin heavy-chain variable genes of the B cell receptor (IGHV). Intermediate-risk prognostic marker includes trisomy 12, and low-risk prognostic factors include 13q and mutated IGHV [2,3].

The clinical presentation of CLL can range from asymptomatic with an indolent course to progressive lymphocytosis, cytopenia, fatigue, hepatosplenomegaly, lymphadenopathy, B-symptoms (i.e., night sweats, weight loss, and fever), autoimmune events (i.e., hemolytic anemia), and recurrent infections. Although rare, cases of ascites have been reported as a clinical manifestation of CLL [4,5]. In these cases, clonal cells found in the ascitic fluid were similar in morphology and immunophenotypically consistent with CLL [2,3,4,5,6]. In these studies, the patients were treated with an alkylating agent such as chlorambucil. Although the use of alkylating agents led to temporary remission of ascites in patients, survival was <1 year in those cases. In our case report, we present the therapeutic management of a patient with CLL presenting with malignant ascites and achieving prolonged survival and reduced occurrence of ascites with the treatment combination of obinutuzumab, a newer more potent anti-CD20 monoclonal antibody, and acalabrutinib, a second generation highly selective Bruton’s tyrosine kinase inhibitor.

## 2. Case Report

The patient is a 73-year-old man with a history of hypertension, type II diabetes mellitus, severe multiple vessel coronary artery disease requiring coronary artery bypass graft (CABG), and asymptomatic aortic valve stenosis who presented with osteomyelitis of the finger, which had been refractory to treatment with oral and intravenous antibiotics and required amputation. During his hospitalization, the patient was noted to have lymphadenopathy and splenomegaly on clinical assessment; abdominal and chest imaging showed lymphadenopathy involving the following lymph nodes: mediastinal, supraclavicular, bilateral axillary, hilar, subpectoral, paraoesophageal, retrocrural, obturator, and external iliac lymph nodes. Abdominal imaging also showed bulky retroperitoneal lymphadenopathy including a 1.1 cm aortocaval and 2.2 cm para-aortic lymph nodes. Imaging showed an enlarged spleen of 18.4 cm in the AP dimension and 16.7 cm in the craniocaudal dimension.

A subsequent biopsy of an inguinal lymph node demonstrated CLL/SLL. Bone marrow aspiration showed 60% lymphocytosis; FISH analysis of bone marrow aspirate showed trisomy for chromosome 12, which is observed in CLL. The patient was treated with six cycles of bendamustine plus rituximab (BR) and achieved complete remission with resolution of lymphadenopathy and organomegaly.

Seven years post remission, the patient began developing recurrent symptomatic ascites requiring therapeutic and diagnostic paracentesis. The peritoneal fluid was transudative with a SAAG (serum-ascites albumin gradient) of 1.8. Diagnostic workup to assess the cause of ascites including abdominal imaging did not reveal evidence of cirrhosis, splenic, or hepatic vein thrombosis. Hepatic function tests were within the normal range. In addition, as the patient had a history of aortic valve stenosis and prior CABG, ruling out right-sided heart failure as an explanation of ascites was essential. Clinically, the patient did not have jugular vein distention or hepato-jugular reflux. Additionally, cardiac biomarkers (brain natriuretic peptide and troponin) were within the normal range, and transthoracic echocardiogram showed grade I diastolic dysfunction with persevered left ventricular ejection fraction (EF ~55–60%).

**Figure 1:**
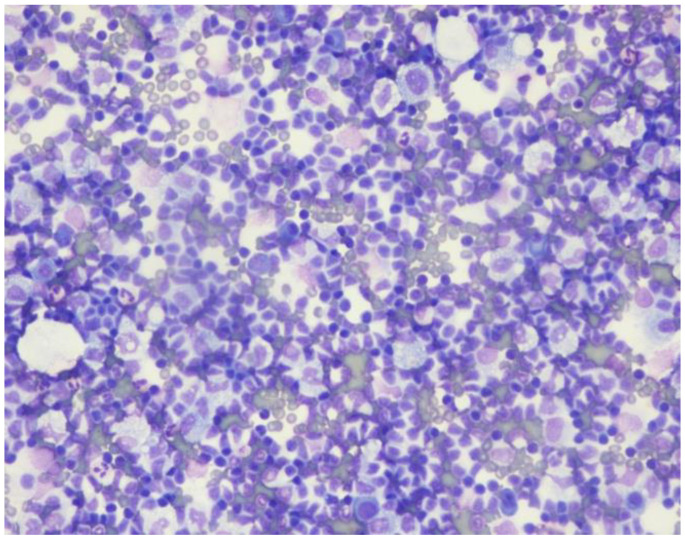
Peritoneal fluid with an increased lymphocyte population.

Ascitic fluid demonstrated increased lymphocytes (Figure 1) and flow cytometry was consistent with CLL (18% aberrant B lymphocytes) positive for CD5, CD19, CD20, CD23 [partial], CD45, lambda light chain [dim], and cytoplasmic Lambda [dim] and negative for CD2, CD3, CD4, CD7, CD8, CD10, CD11b, CD30, CD38, CD43, CD56, CD57, FMC-7, HLA-DR, kappa light chain, and cytoplasmic Kappa. Complete blood count revealed a WBC of 3.9 K, an absolute lymphocyte count of 1 K, and a platelet count of 102 K. Hemoglobin was 12.7 g/dL.

**Figure 2:**
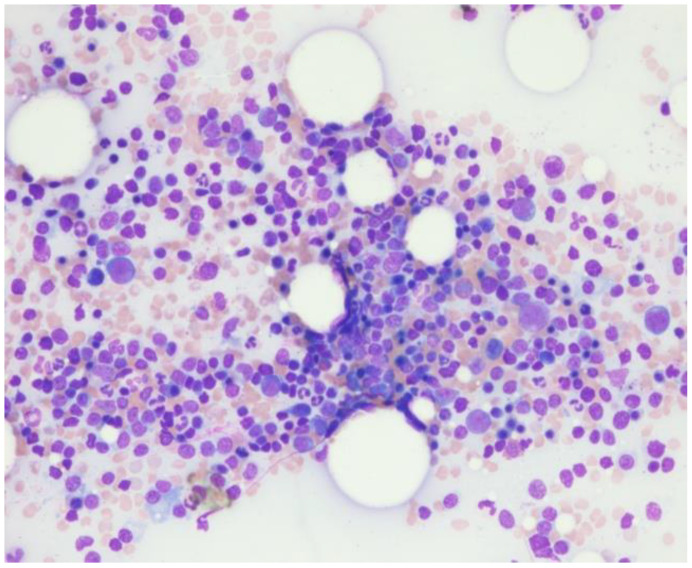
Bone marrow aspirate with trilineage hematopoiesis and increased lymphocytes.

**Figure 3:**
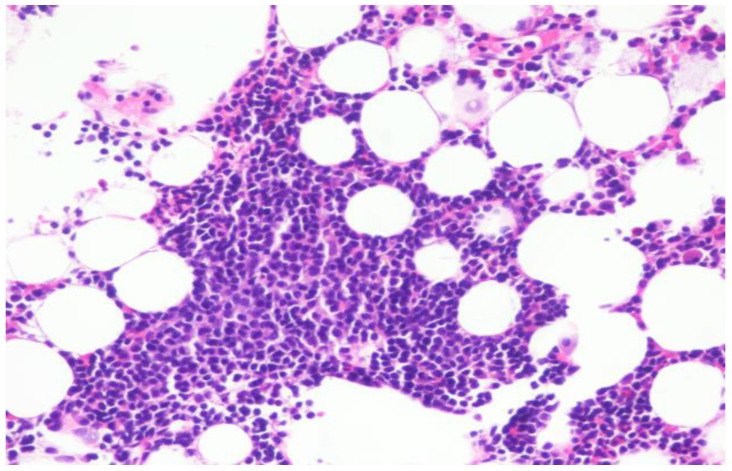
Hypercellular bone marrow with trilineage hematopoiesis as well as increased lymphocytes.

Repeat bone marrow biopsy showed hypercellular marrow with persistent/recurrent involvement by CLL with 35% lymphocytes (Figure 2 and Figure 3). Flow cytometry analysis on bone marrow showed the presence of aberrant clonal B cells (~14% of total events) with the following immunophenotype positive for CD5, CD19, CD20 (dim), CD22, CD23 (subset), CD45, HLA-DR, CD11c (subset), surface Lambda light chain (dim) with a Kappa to Lambda ratio of <1:10 and negative for CD10, CD103, FMC-7, CD38, and surface Kappa light chain. Molecular studies on the bone marrow aspirate did not detect the deletion of 13q, ATM/11q, or TP53 mutation, but was positive for trisomy 12.

**Figure 4:**
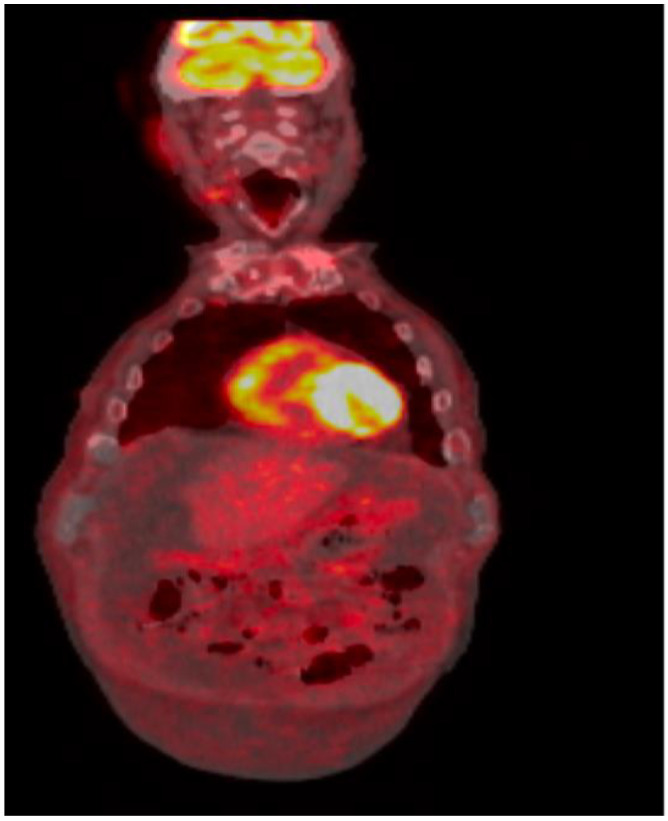
FDG-PET/CT: Large volume ascites with omental caking, the caking exhibited increased FDG activity with an SUV max of 3.1.

Positron emission tomography/computed tomography showed large ascites with omental caking, exhibiting FDG activity with an SUV maximum of 3.1; it also showed mild fluorodeoxyglucose (FDG)-avid right axillary lymph nodes, but no other evidence of the recurrence of CLL (Figure 4).

The patient was initially started on ibrutinib 420 mg orally daily with the addition of rituximab. He received large-volume paracentesis on average every 1–2 weeks. Due to the lack of clinical improvement in ascites, in addition to the lack of tolerance to rituximab (persistent fever and chills despite administering Rituxan at lowest infusion rate at 20 mL/h and high-dose premedication) and ibrutinib (developed atrial fibrillation), the therapy was discontinued after four cycles. The patient developed severe kidney disease requiring multiple sessions of inpatient hemodialysis due to high-volume ascites; the patient required frequent paracentesis with the removal of nearly 9–12 L of fluid with each procedure.

The patient was then transitioned to obinutuzumab. He received 1 g of solumedrol on days 1, 2, 8, and 15 while receiving therapeutic doses of obinutuzumab. The purpose of solumedrol administration was to decrease the risk of infusion reaction to obinutuzumab, as the patient had previously had an adverse infusion reaction to rituximab [7]. High-dose steroids were not continued with obinutuzumab after the first month of immunotherapy. The patient tolerated obinutuzumab without any infusion reactions.

After the first cycle of obinutuzumab, the patient was started on 100 mg of acalabrutinib twice a day; acalabrutinib twice a day was discontinued for a brief period due to severe thrombocytopenia and genitourinary bleeding presenting as hematuria with clots (hemorrhagic events a known side effect). Acalabrutinib was restarted at a decreased frequency of once daily after a month when the platelets recovered to >100,000.

Overall, the patient received six cycles of Obinutuzumab and remained on Acalabrutinib at 100 mg daily.

Prior to starting obinutuzumab, the patient underwent monthly therapeutic paracentesis with the removal of approximately 9–12 L of serous fluid with each procedure. After the second infusion cycle of obinutuzumab, the patient had a dramatic decrease between the intervals of paracentesis performed. A 4-month period passed before the patient underwent therapeutic paracentesis during which ~1 L of serous fluid was removed. The patient has not undergone a therapeutic paracentesis since completing six courses of obinutuzumab. Overall, the patient has had a dramatic improvement and positive outcome in terms of aa reduction in ascites and continued survival after starting obinutuzumab in combination with acalabrutinib.

## 3. Discussion

CLL is the most common hematologic malignancy in the Western world [8]. Although classically associated with indolent disease progression, the clinical course of CLL varies in patients. The spectrum of survival outcomes in patients with CLL ranges from 1–2 years with aggressive variants, all way to 10–20 years among other patients with more indolent courses of disease [6].

Malignant ascites is a clinical presentation associated with certain solid organ tumors such as ovarian cancer, breast cancer, and gastrointestinal malignancies [3]. Ascites presenting as a clinical manifestation of CLL is a rare entity. Only a few case reports have shown patients presenting with ascites at the time of initial diagnosis of CLL or after the initiation and completion of chemotherapy [1,3].

Malignancy-associated ascites in CLL from previous case reports has been characterized as both transudative and exudative peritoneal fluid, with presentation ranging between 18 and 21 months after initiating chemotherapy with chlorambucil. In our case report, the patient presented with transudative ascites requiring diagnostic paracentesis ~7 years after his diagnosis of CLL and the completion of chemotherapy to complete remission. Malignant ascites is an uncommon clinical presentation associated with CLL; thus, providers should be aware that ascites may be associated with the relapse of leukemia and not delay diagnosis. The timing of ascites development will vary in patients; thus, leukemia should be suspected when a patient presents with ascites, in addition to the common clinical features of the disease [3,4,6].

There are certain cases where the peritoneal fluid contains many inflammatory cells and T lymphocytes, thus making the diagnosis of CLL difficult. One study showed a predominance of T cell lymphocytes as opposed to B cells in the ascitic fluid, which was inconsistent with CLL. Other components of the peritoneal fluid in the case reported included a serum ascites albumin gradient of 1.7 and the cytology components included mesothelial cells, macrophages, neutrophils, and >70% small lymphocytes. However, CLL was diagnosed using polymerase chain reaction assays for the clonality analysis of immunoglobulin gene rearrangements of lymphocytes in the ascitic fluid [2,3].

The pathogenesis of ascites in CLL is not well-known. One proposed mechanism is the accumulation of leukemia cells in abdominal lymph nodes and peritoneum; this accumulation incites an inflammatory response, thus leading to the development of ascites [2]. Another hypothesis is the production of vascular endothelial growth factor and vascular permeability factor by lymphoma cells, which increase vascular permeability. Furthermore, simultaneous infiltration of malignant cells in the lymph nodes leads to the blockage of lymphatic channels, thus leading to peritoneal fluid accumulation [9]. Our patient, in this case, developed transudative ascites, where the second hypothesis may be the mechanism by which ascites developed.

Additionally, there may be an association with the type of chemotherapy regimen used and the recurrence of ascites, in addition to the survival of the patient. Previous cases have reported the development of malignant ascites within ≤2 years of initial treatment of CLL with alkylating agents. Although a few of the previous cases have reported the remission of ascites after retreatment with chlorambucil with or without prednisone, the survival outcomes of these patients were not reported, and one case reported the death of the patient 1 month after initiating chemotherapy [3]. In our case, the use of a Bruton’s tyrosine kinase inhibitor, acalabrutinib, combined with an anti-CD20 monoclonal antibody obinutuzumab, resulted in a decreased interval in the development of ascites and a durable response. Our patient has had a prolonged and continued response for >1 year on the regimen described. The combination is well-tolerated and can be used safely in elderly patients with multiple comorbidities, like our patient. Elderly patients have reduced organ function reserve, and the full and therapeutic dosing of chemotherapy is usually not possible, as it is frequently associated with toxicity and reduced tolerability, which eventually leads to decreased therapeutic benefit.

Hence, for patients presenting with ascites as part of CLL, clinicians should consider the use of a combination regimen of obinutuzumab and acalabrutinib (especially in the clinical setting of renal dysfunction and atrial fibrillation where venetoclax and ibrutinib are avoided, respectively) to promote a durable response and survival with decreased adverse effects.

## 4. Conclusions

The presence of ascites can be a rare presenting feature in CLL. The underlying pathology for the rare, atypical presentation with CLL is unknown. Durable response in resolution of recurrent ascites for close to 2 years with newer generation BTK inhibitor- acalabrutinib and anti-CD20 monoclonal antibody, obinutuzumab is achievable despite past failure to first generation BTK inhibitor, ibrutinib, and the anti-CD20 monoclonal antibody rituxan.

## Data Availability

Not applicable.

## Abbreviations

BR	Bendamustine plus rituximab
CLL	Chronic lymphocytic leukemia
FISH	Fluorescence in situ hybridization
IGHV	Immunoglobulin heavy-chain variable gene
MBL	Malignant B lymphocytes
SLL	Small lymphocytic leukemia
TTE	Transthoracic echocardiogram
VPF	Vascular permeability factor
VEGF	Vascular endothelial growth factor
